# Gastrointestinal stromal tumors with the use of ripretinib and sunitinib: real-world adverse event analysis based on the FDA adverse event reporting system (FAERS)

**DOI:** 10.3389/fphar.2025.1561937

**Published:** 2025-06-26

**Authors:** Xinzhen Che, Yong Zhu

**Affiliations:** ^1^ First Clinical Medical College, Shandong University of Traditional Chinese Medicine, Jinan, Shandong, China; ^2^ Shandong University of Traditional Chinese Medicine Affiliated Hospital, Jinan, Shandong, China

**Keywords:** ripretinib, sunitinib, adverse events, FAERS, GIST treatment

## Abstract

**Objective:**

This study aims to analyze potential adverse events (AEs) associated with ripretinib and sunitinb in gastrointestinal stromal tumor (GIST) treatment using data from the FDA Adverse Event Reporting System (FAERS). The findings provide insights for future research to improve the safety and clinical management of ripretinib and sunitinib.

**Methods:**

Adverse Drug Event (ADE) reports related to ripretinib and sunitinib were extracted from the FAERS database, covering the period from Q2 2020 to Q4 2024 and Q1 2006 to Q4 2024, respectively. ADEs were classified and described according to Preferred Terms (PTs) and System Organ Classes (SOCs) in the Medical Dictionary for Regulatory Activities (MedDRA). Disproportionality analysis, including Reporting Odds Ratio (ROR), Proportional Reporting Ratio (PRR), Bayesian Confidence Propagation Neural Network (BCPNN), and Multi-Item Gamma Poisson Shrinker (MGPS), was employed to identify significant signals.

**Results:**

A total of 3,636 and 34,768 ADE reports related to ripretinib and sunitinib were identified using four disproportionality analysis methods. The top five ADR signals for ripretinib include hepatic embolization, tumor compression, hyperkeratosis, tumor excision and tumor pain. For sunitinib, the five strongest ADR signals are metastatic renal cell carcinoma, diffuse uveal melanocytic proliferation, renal cancer metastasis, connective tissue neoplasm and salivary gland fistula. Both drugs share significant ADRs including palmar-plantar erythrodysesthesia syndrome, disease progression and hyperkeratosis. Furthermore, subgroup analysis was conducted to explore sex difference in ripretinib and sunitinib.

**Conclusion:**

This study validated known AEs and identified new potential safety signals associated with ripretinib and sunitinib in GIST treatment. These findings contribute to the understanding of ripretinib and sunitinib, providing valuable evidence for improving its clinical use.

## 1 Introduction

Gastrointestinal stromal tumor (GIST) is the most common mesenchymal tumor of the gastrointestinal tract, primarily affecting the stomach and small intestine, with an estimated global incidence of 10–15 cases per million individuals (Søreide et al., 2016) (Kelly et al., 2021). Targeted cancer therapies have emerged as a significant advancement in prolonging the survival of patients with advanced GIST (3). Approximately 80% of GIST cases harbor activating mutations in the stem cell factor receptor (KIT) receptor tyrosine kinase gene, while 5%–10% involve mutations in the platelet-derived growth factor receptor alpha (PDGFRA) gene (Dhillon, 2020). Identifying the molecular subtypes of GIST is of critical importance in clinical treatment. Although imatinib is highly effective as first-line therapy for GISTs, secondary resistance mutations frequently arise, necessitating subsequent second-line treatment with sunitinib (Cohen et al., 2021). Sunitinib, an oral multi-targeted tyrosine kinase inhibitor (TKI), mediates antitumor activity by simultaneously inhibiting angiogenesis and tumor cell proliferation through targeting vascular endothelial growth factor receptors (VEGFRs), platelet-derived growth factor receptors (PDGFRs), and KIT (6).

In GIST patients exhibiting progression after sequential treatment with imatinib and sunitinib, tumor cells develop complex kinase conformational alterations via acquired mutations, significantly reducing the efficacy of conventional TKIs. This clinical challenge prompted the development of novel targeted therapies, notably ripretinib, a breakthrough fourth-line agent. Ripretinib, the first FDA-approved broad-spectrum kinase inhibitor for fourth-line GIST treatment (Ripretinib for gastrointestinal stromal tumours, 2021), uniquely targets both the kinase activation loop and switch pocket, effectively suppressing diverse resistance mutations including KIT and PDGFRA variants (Goggin et al., 2022).

In May 2020, ripretinib received FDA approval based on results from the Phase III INVICTUS trial, indicated for advanced GIST patients with disease progression following previous treatments with imatinib, sunitinib, and regorafenib. Its favorable safety profile and enhanced tolerability facilitate prolonged disease control in advanced stages, representing a significant advancement in addressing TKI resistance. Although Ripretinib demonstrates favorable safety and tolerability profiles in clinical trials, adverse events (AEs) remain inevitable in real-world applications. In the phase III INVICTUS trial, the most common AEs (incidence >20%) included alopecia, myalgia, nausea, fatigue, palmar–plantar erythrodysesthesia, and diarrhoea (Blay et al., 2020). Identifying these AEs is crucial for ensuring patient safety and optimizing clinical outcomes, necessitating the application of data mining algorithms to detect potential safety signals of Ripretinib in real-world settings.

The U.S. Food and Drug Administration Adverse Event Reporting System (FAERS) is one of the largest post-marketing safety monitoring databases, and the reliability and validity of its data have been widely recognized in the industry (Chen et al., 2021; Yu et al., 2021). Using the FAERS database, pharmacovigilance research related to ripretinib and sunitinib was conducted to identify adverse events (AEs) not described on the drug label. These findings not only enhance research on ripretinib by comparing classical drug-sunitinib, providing valuable references for clinical drug use.

## 2 Materials and methods

### 2.1 Data source

**FIGURE 1:**
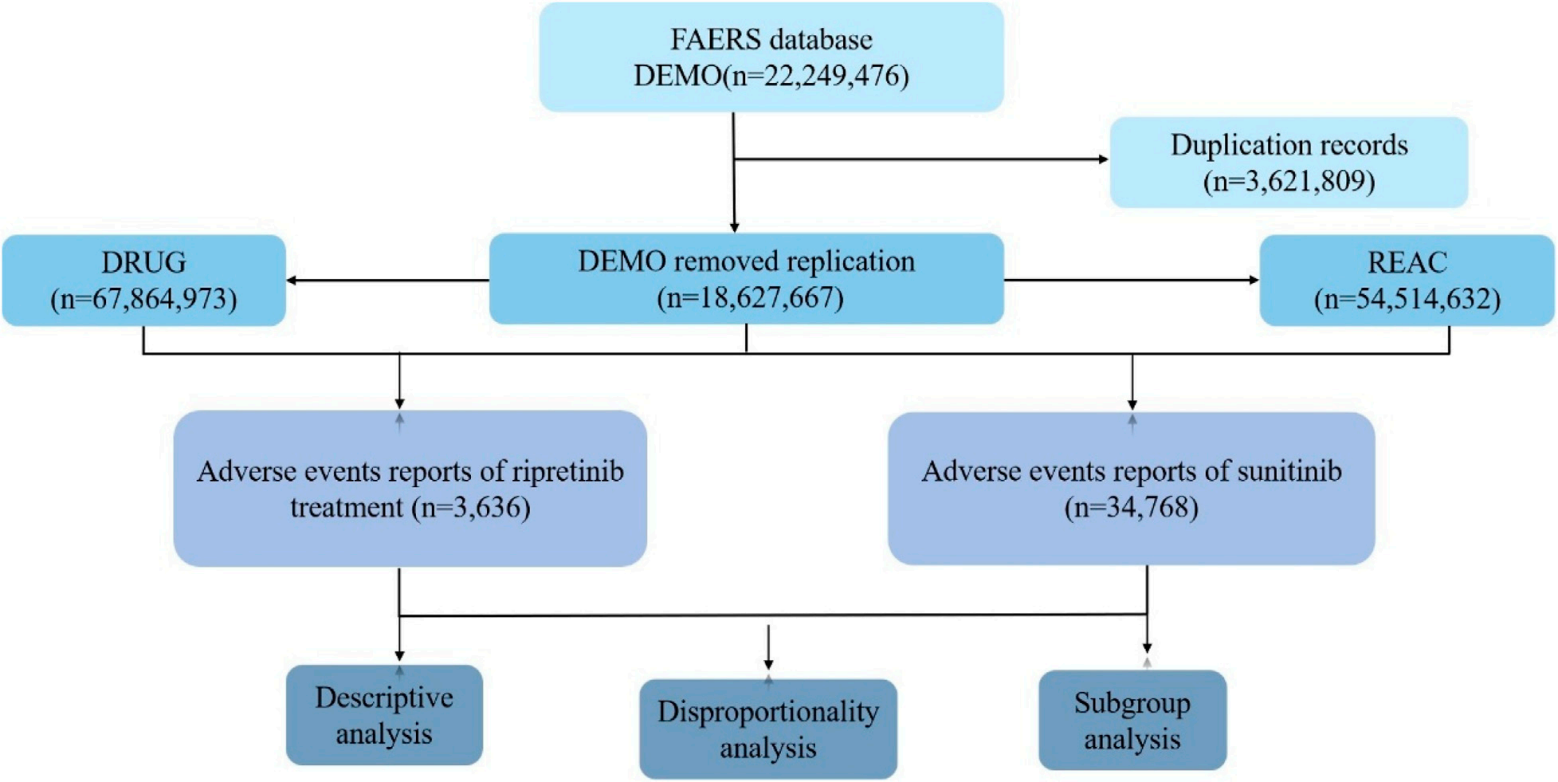
Flowchart of ripretinib and sunitinib in FAERS.

The FAERS database is a publicly accessible, voluntary, and spontaneous reporting system designed for post-marketing surveillance of FDA-approved drugs. It collects adverse drug event (ADE) reports from healthcare professionals, patients, and pharmaceutical manufacturers worldwide, reflecting real-world ADE occurrences (Guo et al., 2022; Pan et al., 2024). This study aims to systematically evaluate the post-marketing safety of ripretinib and sunitinib (Figure 1). Only reports of ripretinib and sunitinib as primary suspect drugs were included. For cases with duplicate CASEIDs, the record with the most recent FDA_DT or the highest PRIMARYID was retained. Subsequently, AEs were standardized, classified, and described based on the Preferred Terms (PT) and System Organ Classes (SOC) defined in the Medical Dictionary for Regulatory Activities (MedDRA 26.1). Some of the analysis results were computed using R software 4.4.3, with packages including “dplyr”, “ggplot2″, “forestplot” and “data.table” for data handling and visualization.

Since the FAERS database is publicly accessible and patient records are anonymized and de-identified, this study does not involve informed consent or ethical approval.

### 2.2 Methods of data analysis

Disproportionality analysis is a tool used to generate hypotheses about specific drugs and AEs and to clinically evaluate potential case reports (Zh et al., 2025). Four methods were employed for ADE signal mining, including Reporting Odds Ratio (ROR), Proportional Reporting Ratio (PRR), Bayesian Confidence Propagation Neural Network (BCPNN), and Multi-item Gamma Poisson Shrinker (MGPS) (Luo et al., 2025). The ROR and PRR algorithms are non-Bayesian methods, with the advantage of ROR being its ability to correct bias when event reports are limited, while the advantage of PRR is lower sensitivity by the omission of certain AEs (Li et al., 2025). The MHRA method extends PRR by combining PRR values with absolute report numbers and chi-square values, ensuring a minimum number of case combinations (Zhou et al., 2023). The BCPNN method can perform early signal detection even with limited or missing data, and as the number of reports increases, the detection results become more stable (Godfrey et al., 2025). Compared to non-Bayesian algorithms, Bayesian algorithms have higher specificity, signal stability, and lower misclassification probabilities. The data analysis process in this study is illustrated in Figure 1. The formulas and signal detection criteria of the four methods refer to the methods given in previous literature (Xiong et al., 2023; Kuai et al., 2024). In this study, AE signals were considered significant only if they met the criteria for all four algorithms simultaneously.

## 3 Results

### 3.1 General analysis of AEs in ripretinib and sunitinib

**FIGURE 2:**
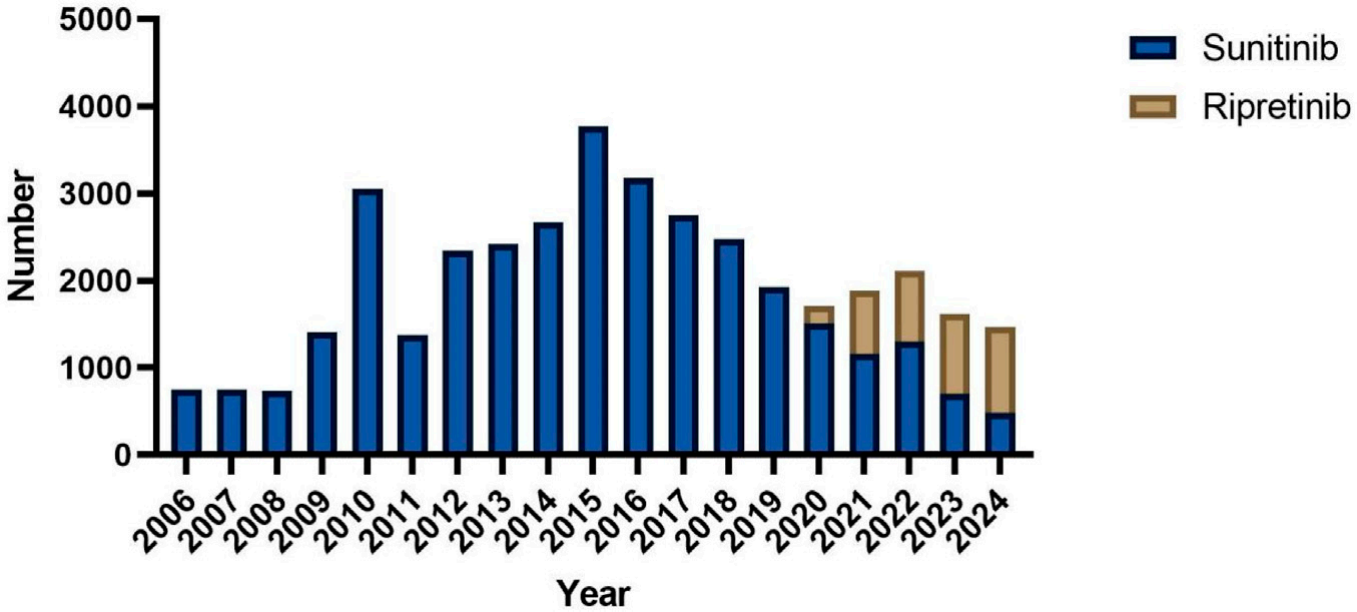
Comparation of reporting year between snuitinib and ripretinib.

A total of 3,636 and 34,768 individual AE reports for ripretinib and sunitinib, respectively, were extracted from the FAERS database (Table 1). In terms of gender distribution, 54.2% of ripretinib-related reports were male, compared to 59.3% in the sunitinib group. Female patients accounted for 43.7% in the ripretinib group and 31.8% in the sunitinib group. Age information was missing in 52.0% of ripretinib cases and 24.8% of sunitinib cases. Among the available data, patients aged ≥65 years comprised the largest proportion in both groups (29.5% for ripretinib and 38.1% for sunitinib), followed by those aged 18–64 years (18.4% vs 36.9%). Reports involving patients under 18 years were rare in both groups. Weight data were largely incomplete, with 91.1% missing in the ripretinib group and 73.4% in the sunitinib group. Among the reports with available weight, most patients weighed between 50 and 100 kg in both groups. Regarding reporter type, most ripretinib cases were submitted by consumers (61.4%), whereas sunitinib cases were more frequently reported by physicians (29.9%) and consumers (38.7%). Reports from other healthcare professionals, such as pharmacists and non-physician health professionals, were more prevalent in the sunitinib group. The largest proportion of outcomes reported for ripretinib was categorized as “hospitalization,” accounting for 18.2% of the total cases expect missing cases. “other outcomes” and “death” were also important outcomes, comprising 11.4% and 10.6% of total reports. For sunitinib, death was the most frequent outcome (29.8% of cases), followed by “other outcomes” (22.1%) and “hospitalization” (21.3%). Geographically, the majority of ripretinib reports originated from the United States (93.1%), whereas sunitinib reports were more geographically diverse, with 49.1% from the United States, followed by notable contributions from Japan (5.6%), Argentina (5.3%), China (5.2%), and India (4.0%). The annual distribution of reports revealed distinct patterns between sunitinib and ripretinib. Sunitinib-related reports have been submitted consistently since 2006, with a marked increase beginning in 2010 and peaking around 2016. After 2017, the number of reports gradually declined. In contrast, ripretinib-related reports only appeared from 2020 onwards, aligning with its later market approval. Since then, the number of ripretinib reports has shown a steady increase, surpassing sunitinib in total annual reports by 2022 (Figure 2).

**TABLE 1:** Clinical characteristics of patients with GIST in sunitinib and ripretinib from the FAERS database.

Characteristics	Ripretinib	Sunitinib
Number of reports	3636	34,768
Gender		
Male	1969 (54.2%)	20,608 (59.3%)
Female	1589 (43.7%)	11,072 (31.8%)
Missing	78 (2.1%)	3088 (8.9%)
Age (years), n (%)		
<18	2 (0.1%)	63 (0.2%)
18–64	670 (18.4%)	12,821 (36.9%)
≥65	1074 (29.5%)	13,263 (38.1%)
Missing	1890 (52.0%)	8621 (24.8%)
Weight (kg), n (%)		
<50	39 (1.1%)	559 (1.6%)
50–100	252 (6.9%)	7631 (21.9%)
>100	31 (0.9%)	1054 (3.0%)
Missing	3314 (91.1%)	25,524 (73.4%)
Reporter, n (%)		
Consumer	2232 (61.4%)	13,449 (38.7%)
Health Professional	715 (19.7%)	939 (2.7%)
Physician	598 (16.4%)	10,399 (29.9%)
Other Professional	0	5915 (17.0%)
Pharmacist	86 (2.4%)	2867 (8.2%)
Missing	5 (0.1%)	1199 (3.5%)
Outcome, n (%)		
Death	384 (10.6%)	10,353 (29.8%)
Hospitalization	662 (18.2%)	7422 (21.3%)
Life threatening	11 (0.3%)	767 (2.2%)
Disability	0	145 (0.4%)
Other outcomes	415 (11.4%)	7657 (22.1%)
Missing	2164 (59.5%)	8424 (24.2%)
Country, n (%)		
The United States	3385 (93.1%)	17,065 (49.1%)
France	60 (1.7%)	
Canada	46 (1.3%)	
The United Kingdom	14 (0.4%)	
Australia	13 (0.4%)	
Japan		1947 (5.6%)
Argentina		1837 (5.3%)
China		1794 (5.2%)
India		1390 (4.0%)

### 3.2 Signal detection at the SOC level

**FIGURE 3:**
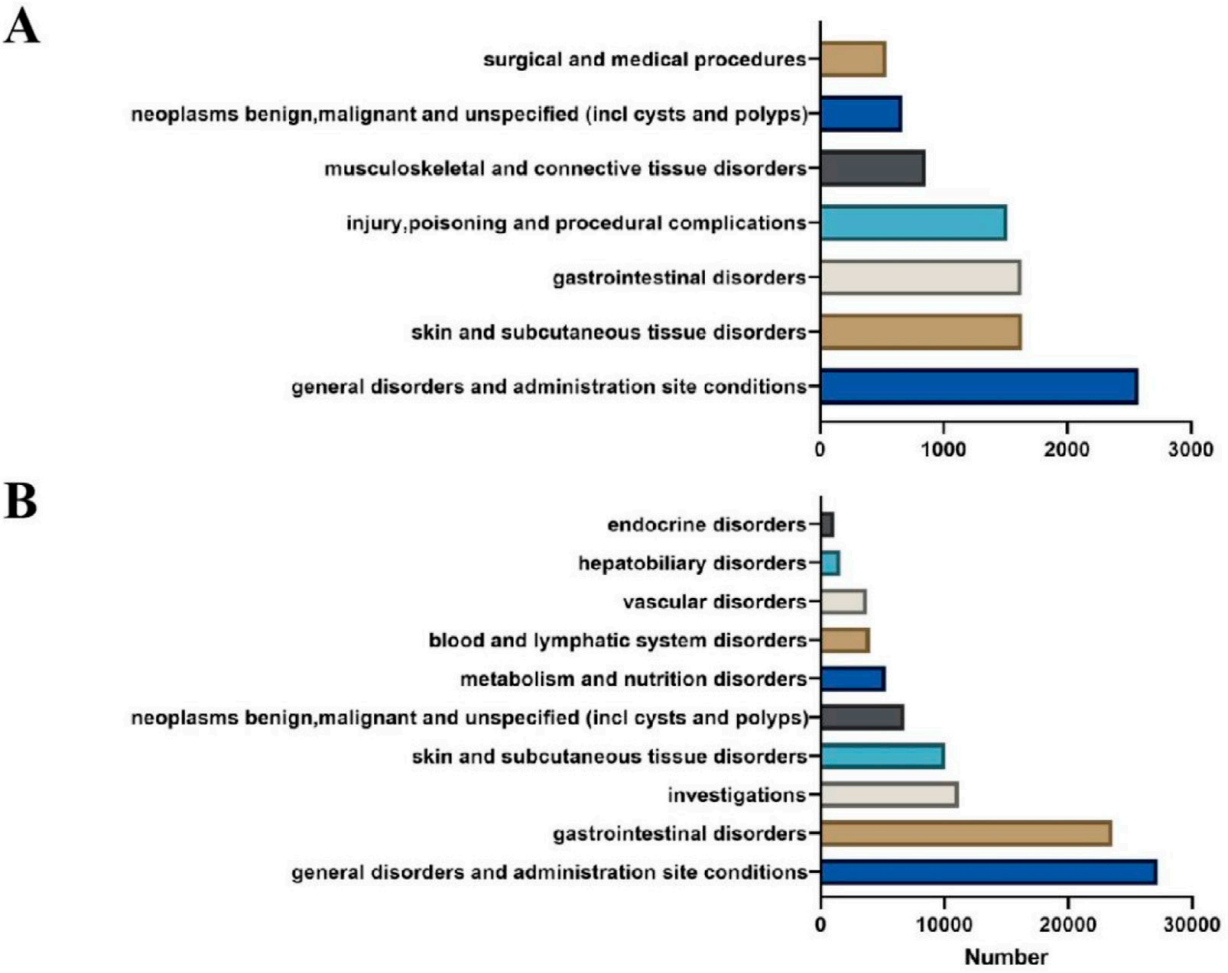
Comparation between ripretinib and sunitinib at the SOC level. **(A)** AE signals at the SOC level in ripretinib. **(B)** AE signals at the SOC level in sunitinib.

Ripretinib and snuitinib related AEs were associated with 27 distinct system organ classes (SOCs), of which 7 and 10 met the criteria of all four disproportionality analysis methods (Figure 3). Among all SOCs, the most commonly reported in ripretinib were general disorders and administration site conditions (n = 2579), skin and subcutaneous tissue disorders (n = 1637) and gastrointestinal disorders (n = 1632). General disorders and administration site conditions (n = 27,124), gastrointestinal disorders (n = 23,592) and investigations (n = 11,178) were most frequent SOCs in snuitinb, which mostly consistent with ripretinib. Moreover, as for ripretinib, attention should also be paid to less common SOCs such as musculoskeletal and connective tissue disorders and neoplasms benign, malignant and unspecified (incl cysts and polyps) in clinical practice.

### 3.3 Signal detection at the PT level

Based on disproportionality analysis, both ripretinib and sunitinib exhibit significant ADR signals in Table 2, 3. The top five ADR signals for ripretinib include hepatic embolization (ROR = 177.62, 95%CI: 55.92–564.17), tumor compression (ROR = 159.5, 95%CI: 87.32–291.34), hyperkeratosis (ROR = 90.43, 95%CI: 74.54–109.7), tumor excision (ROR = 82.25, 95%CI: 48.45–139.64), and tumor pain (ROR = 74.04, 95%CI: 48.08–114.03). For sunitinib, the five strongest ADR signals are metastatic renal cell carcinoma (ROR = 270.16, 95%CI: 245.71–297.04), diffuse uveal melanocytic proliferation (ROR = 133.8, 95%CI: 43.15–414.88), renal cancer metastasis (ROR = 127.34, 95%CI: 107.62–150.66), connective tissue neoplasm (ROR = 109.48, 95%CI: 44.39–270.01), and salivary gland fistula (ROR = 109.48, 95%CI: 30.54–392.42).

**TABLE 2:** Top 30 AE analysis ranked by ROR value in ripretinib

PT	N	ROR (95%Cl)	PRR (χ^2^)	EBGM(EBGM05)	IC(IC025)
Hepatic embolisation	3	177.62 (55.92–564.17)	177.58 (505.1)	170.32 (64.76)	7.41 (5.93)
Tumour compression	11	159.5 (87.32–291.34)	159.36 (1666.95)	153.5 (92.72)	7.26 (6.41)
Hyperkeratosis	106	90.43 (74.54–109.7)	89.71 (9101.86)	87.83 (74.72)	6.46 (6.17)
Tumour excision	14	82.25 (48.45–139.64)	82.16 (1100.66)	80.59 (51.75)	6.33 (5.58)
Tumour pain	21	74.04 (48.08–114.03)	73.93 (1484.3)	72.65 (50.62)	6.18 (5.56)
Ephelides	8	69.83 (34.71–140.47)	69.78 (533.41)	68.64 (38.25)	6.1 (5.13)
Scan abnormal	4	50.85 (18.97–136.34)	50.84 (193.07)	50.24 (22.01)	5.65 (4.35)
Extra dose administered	347	48.44 (43.51–53.92)	47.18 (15,517.6)	46.66 (42.66)	5.54 (5.39)
Gastric neoplasm	6	43.71 (19.55–97.73)	43.69 (247.68)	43.25 (22.06)	5.43 (4.34)
Product coating issue	9	42.36 (21.96–81.7)	42.33 (359.5)	41.91 (24.19)	5.39 (4.47)
Abdominal cavity drainage	4	41.76 (15.59–111.83)	41.75 (157.5)	41.34 (18.13)	5.37 (4.07)
Neoplasm progression	244	31.03 (27.33–35.24)	30.47 (6909.28)	30.26 (27.21)	4.92 (4.73)
Skin hypertrophy	17	28.23 (17.52–45.5)	28.2 (442.97)	28.01 (18.79)	4.81 (4.13)
Acrochordon	8	28.06 (14–56.25)	28.04 (207.23)	27.86 (15.57)	4.8 (3.83)
Melanocytic naevus	27	27.94 (19.13–40.81)	27.88 (695.22)	27.7 (20.18)	4.79 (4.24)
Palmar-plantar Erythrodysaesthesia syndrome	136	27.05 (22.83–32.04)	26.78 (3354.45)	26.61 (23.09)	4.73 (4.49)
Cancer pain	14	25.96 (15.34–43.91)	25.93 (333.5)	25.78 (16.6)	4.69 (3.94)
Nipple disorder	3	21.18 (6.81–65.87)	21.18 (57.38)	21.07 (8.15)	4.4 (2.95)
Spinal cord neoplasm	4	20.78 (7.78–55.5)	20.77 (74.89)	20.67 (9.08)	4.37 (3.07)
Biopsy	4	20.67 (7.74–55.22)	20.67 (74.48)	20.57 (9.04)	4.36 (3.07)
Abdominal neoplasm	4	20.65 (7.73–55.15)	20.64 (74.38)	20.54 (9.03)	4.36 (3.07)
Nutritional condition abnormal	3	20.22 (6.5–62.86)	20.21 (54.52)	20.12 (7.79)	4.33 (2.88)
Hepatic neoplasm	14	19.67 (11.63–33.26)	19.65 (246.66)	19.56 (12.6)	4.29 (3.54)
Solar lentigo	4	19.25 (7.21–51.43)	19.25 (68.89)	19.16 (8.42)	4.26 (2.97)
Underdose	286	18.7 (16.63–21.03)	18.31 (4666.06)	18.24 (16.53)	4.19 (4.02)
Skin papilloma	16	18.05 (11.04–29.5)	18.03 (256.26)	17.96 (11.9)	4.17 (3.46)
Metastases to peritoneum	9	17.49 (9.08–33.66)	17.47 (139.2)	17.41 (10.06)	4.12 (3.21)
Product shape issue	3	14.68 (4.72–45.61)	14.68 (38.1)	14.63 (5.66)	3.87 (2.42)
Hair texture abnormal	45	14.6 (10.89–19.57)	14.55 (565.92)	14.5 (11.35)	3.86 (3.43)
Oncologic complication	3	14.13 (4.55–43.9)	14.13 (36.46)	14.08 (5.45)	3.82 (2.37)

**TABLE 3:** Top 30 AE analysis ranked by ROR value in sunitinib.

PT	N	ROR (95%Cl)	PRR (χ^2^)	EBGM(EBGM05)	IC(IC025)
Metastatic renal cell carcinoma	715	270.16 (245.71–297.04)	268.73 (114,239.39)	161.37 (149.05)	7.33 (7.21)
Diffuse uveal melanocytic proliferation	4	133.8 (43.15–414.88)	133.8 (395.42)	100.6 (39.03)	6.65 (5.18)
Renal cancer metastatic	179	127.34 (107.62–150.66)	127.17 (17,016.14)	96.81 (84.1)	6.6 (6.36)
Connective tissue neoplasm	6	109.48 (44.39–270.01)	109.47 (506.7)	86.23 (40.51)	6.43 (5.21)
Salivary gland fistula	3	109.48 (30.54–392.42)	109.47 (253.35)	86.23 (29.63)	6.43 (4.81)
Renal cell carcinoma stage iv	23	101.47 (64.22–160.33)	101.45 (1826.11)	81.19 (55.37)	6.34 (5.7)
Haemangiopericytoma	4	100.35 (33.55–300.18)	100.35 (314.75)	80.48 (32.18)	6.33 (4.89)
Renal cell carcinoma	896	98.62 (91.66–106.11)	97.97 (69,133.64)	78.95 (74.25)	6.3 (6.2)
Yellow skin	640	71.24 (65.49–77.5)	70.91 (37,490.48)	60.41 (56.3)	5.92 (5.79)
Malignant urinary tract neoplasm	16	69.82 (41.06–118.72)	69.81 (924.41)	59.62 (38.23)	5.9 (5.15)
Eyelash discolouration	29	65.78 (44.42–97.42)	65.77 (1589.28)	56.65 (40.78)	5.82 (5.26)
Gastrointestinal stromal tumour	312	57.76 (51.3–65.05)	57.63 (15,183.73)	50.52 (45.74)	5.66 (5.49)
Alveolar soft part sarcoma	4	47.23 (16.76–133.08)	47.22 (161.93)	42.36 (17.8)	5.4 (4.03)
Scrotal inflammation	5	40.96 (16.32–102.8)	40.96 (176.87)	37.26 (17.25)	5.22 (3.98)
Thyroid atrophy	6	40.82 (17.63–94.55)	40.82 (211.56)	37.14 (18.39)	5.22 (4.07)
Pancreatic neuroendocrine tumour	50	38.46 (28.77–51.41)	38.45 (1664.32)	35.17 (27.59)	5.14 (4.71)
Anal injury	13	35.99 (20.41–63.48)	35.99 (405.82)	33.11 (20.59)	5.05 (4.24)
Plantar erythema	17	32.19 (19.64–52.76)	32.19 (475.59)	29.87 (19.76)	4.9 (4.19)
Thymic cancer metastatic	3	31.69 (9.78–102.66)	31.69 (82.64)	29.44 (11.01)	4.88 (3.37)
Palmar-plantar erythrodysaesthesia syndrome	1356	27.73 (26.23–29.3)	27.46 (32,368.06)	25.76 (24.6)	4.69 (4.61)
Pancreatic neuroendocrine Tumour metastatic	14	27.42 (15.95–47.11)	27.41 (333.51)	25.72 (16.35)	4.69 (3.92)
Myxoedema	12	26.47 (14.76–47.47)	26.47 (275.86)	24.89 (15.27)	4.64 (3.81)
Thymoma malignant	4	25.9 (9.42–71.19)	25.9 (89.94)	24.39 (10.47)	4.61 (3.27)
Neoplasm progression	1857	23.93 (22.83–25.09)	23.62 (38,009.99)	22.36 (21.5)	4.48 (4.41)
Mouth injury	78	23.9 (19.01–30.03)	23.88 (1614.08)	22.6 (18.66)	4.5 (4.16)
Tumour rupture	26	23.62 (15.9–35.08)	23.61 (531.74)	22.36 (16.06)	4.48 (3.91)
Oral pain	1071	22.19 (20.86–23.6)	22.02 (20,381.52)	20.93 (19.87)	4.39 (4.3)
Hyperkeratosis	221	18.58 (16.24–21.27)	18.55 (3508.64)	17.78 (15.88)	4.15 (3.95)
Cardiopulmonary failure	150	17.17 (14.58–20.22)	17.15 (2187.79)	16.49 (14.38)	4.04 (3.8)
Jaw fistula	5	16.87 (6.89–41.27)	16.87 (71.62)	16.23 (7.67)	4.02 (2.81)

Both drugs share significant ADRs including palmar-plantar erythrodysesthesia syndrome (Ripretinib: ROR = 27.05; Sunitinib: ROR = 27.73), disease progression (Ripretinib: ROR = 31.03; Sunitinib: ROR = 23.93), and hyperkeratosis (Ripretinib: ROR = 90.43; Sunitinib: ROR = 18.58), indicating possible similarities in pharmacological effects or biological mechanisms.

However, significant differences exist in the ADR profiles between the two drugs. Ripretinib tends to cause tumor-associated direct complications such as tumor compression and surgical-related reactions, while Sunitinib predominantly involves metastasis of specific tumor types and rare pathological conditions like renal cancer metastasis and uveal melanocytic proliferation. Consequently, individualized risk management strategies should be implemented based on their distinct ADR characteristics in clinical practice.

### 3.4 Gender-based differences in AEs

**FIGURE 4:**
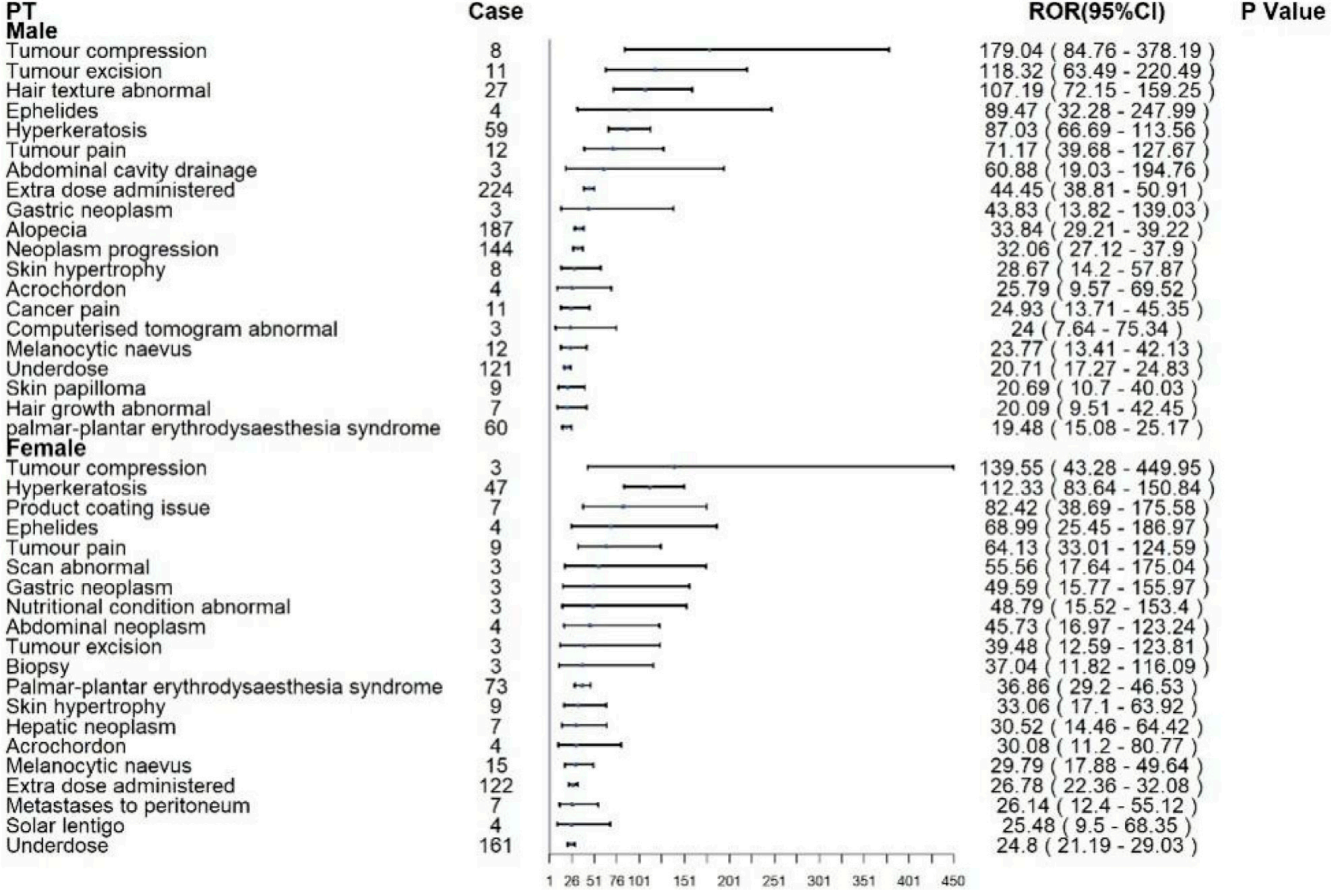
Subgroup analysis at the PT level of ripretinib.

Subgroup analyses were conducted to identify sex-specific patterns of AEs associated with ripretinib and sunitinib. For ripretinib in Figure 4, male patients most frequently reported alopecia (n = 187), extra dose administered (n = 224), and neoplasm progression (n = 144), with significant disproportionality signals (RORs: 33.84, 44.45, and 32.06, respectively). Notably, tumor compression (ROR: 179.04; 95% CI: 84.76–378.19), tumor excision (ROR: 118.32), and hair texture abnormal (ROR: 107.19) showed the strongest associations. Other relevant AEs included hyperkeratosis (n = 59, ROR: 87.03) and palmar-plantar erythrodysaesthesia syndrome (n = 60, ROR: 19.48).Among females, the top reported AEs were underdose (n = 161), extra dose administered (n = 122), and palmar-plantar erythrodysaesthesia syndrome (n = 73). Tumor compression (ROR: 139.55; 95% CI: 43.28–449.95), hyperkeratosis (ROR: 112.33), and product coating issues (ROR: 82.42) demonstrated strong disproportionality.

**FIGURE 5:**
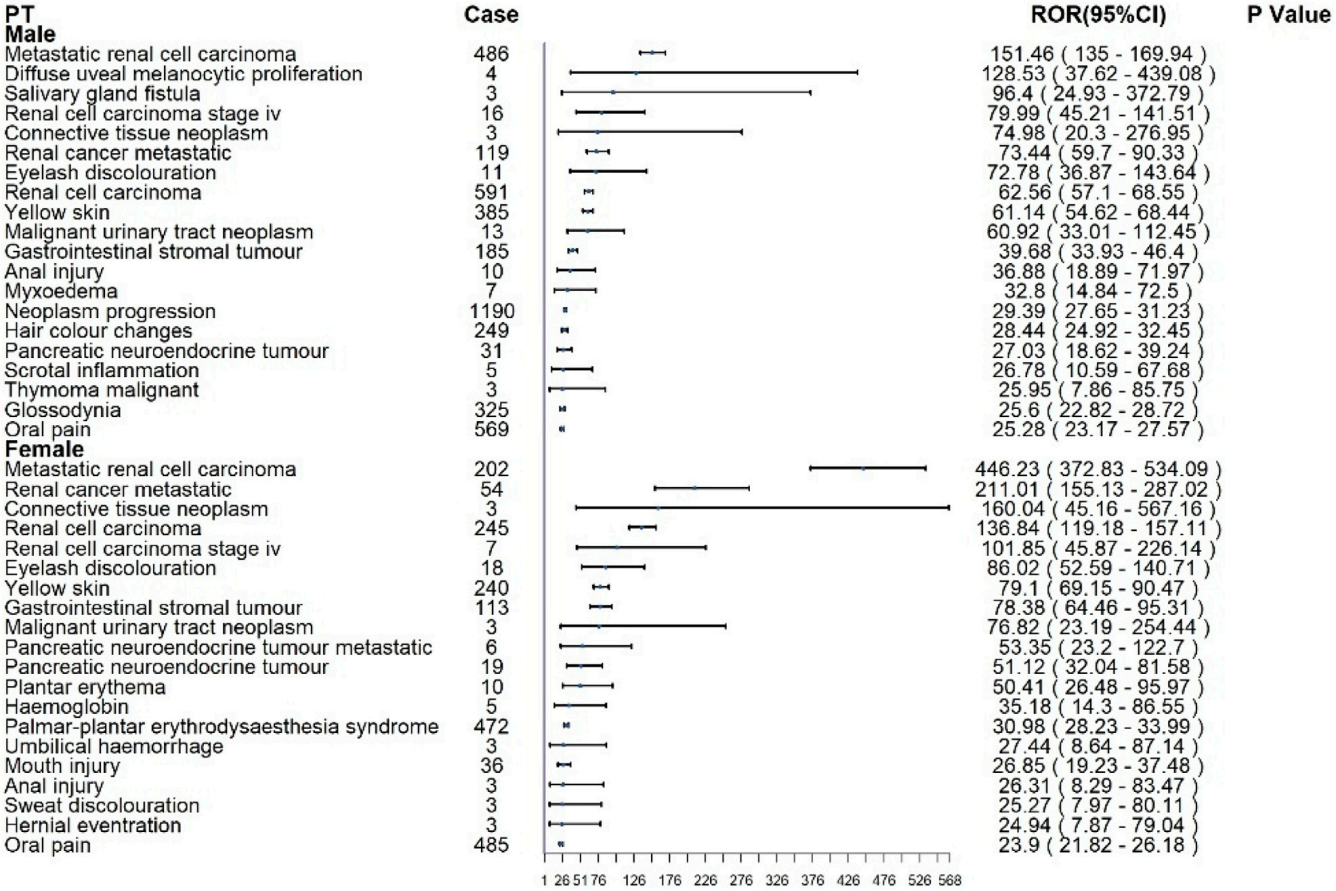
Subgroup analysis at the PT level of sunitinib.

For sunitinib in Figure 5, males most commonly reported renal cell carcinoma (n = 591), metastatic RCC (n = 486), neoplasm progression (n = 1,190), and oral pain (n = 569). The strongest disproportionality was observed for metastatic RCC (ROR: 151.46; 95% CI: 135.0–169.94) and diffuse uveal melanocytic proliferation (BDUMP) (ROR: 128.53; 95% CI: 37.62–439.08), the latter being a rare but fatal ocular event. In female patients, renal cell carcinoma (n = 245), metastatic RCC (n = 202), and oral pain (n = 485) were the most frequent. Disproportionality was greatest for metastatic RCC (ROR: 446.23; 95% CI: 372.83–534.09), renal cancer metastatic (ROR: 211.01), and connective tissue neoplasm (ROR: 160.04).

## 4 Discussion

The ADR analysis derived from the FAERS database reveal critical similarities and differences between ripretinib and sunitinib, two widely used multi-target TKIs. Expanding upon previous studies that examined ripretinib individually, this analysis includes a comparative approach incorporating subgroup analysis to provide a more comprehensive evaluation of their ADR characteristics.

Both ripretinib and sunitinib exhibited overlapping ADRs, predominantly palmar-plantar erythrodysesthesia syndrome (PPES), hyperkeratosis, and paradoxical disease progression. Mechanistic analysis suggests that PPES may originate from potent VEGFR signaling inhibition by both agents, leading to microvascular dysfunction and subsequent inflammatory tissue damage (Zhang et al., 2023). Notably, a multicenter phase III trial revealed significantly higher PPES incidence in the treatment group compared with placebo (12.5% vs. 0.8%), establishing it as the second most frequent ADR following hypertension (Lin et al., 2023), which corroborates our findings. The comparable hyperkeratosis rates observed with both TKIs imply a class effect potentially mediated through EGFR or PDGFR pathway inhibition, which may disrupt keratinocyte proliferation and differentiation homeostasis (Malovitski et al., 2023). However, current pharmacovigilance data present an apparent discrepancy: while case report document ripretinib-associated hyperkeratosis (Muskat et al., 2022), no published clinical studies explicitly link sunitinib to this dermatological manifestation. This comparative analysis provides clinical evidence that hyperkeratosis represents a TKI-class adverse effect rather than a ripretinib-specific phenomenon. Of particular clinical significance was the identification of disease progression as an ADR, which likely reflects treatment failure mechanisms involving secondary kinase mutations that confer therapeutic resistance or insufficient target inhibition. This paradoxical phenomenon underscores the need for molecular monitoring during TKI therapy escalation.

Subgroup analysis by gender offers critical insights into drug-specific ADRs. For ripretinib, both male and female patients exhibited elevated risks of tumor compression, hyperkeratosis, and tumor-related surgical interventions. Notably, female patients demonstrated higher incidences of tumor-associated pain and abnormal imaging findings, while males were more frequently affected by direct tumor-related complications, such as tumor excision and abdominal drainage. These disparities may reflect sex-specific pharmacokinetic and pharmacodynamic profiles, including hormonal influences and differences in metabolic pathways. In contrast, gender differences in sunitinib-related ADRs were more pronounced. Male patients showed higher frequencies of metastatic renal cell carcinoma, salivary gland fistula, and eyelash discoloration, whereas females exhibited increased risks of renal cancer metastasis and yellow skin pigmentation. The ocular and cutaneous toxicities are likely attributable to sunitinib’s potent inhibition of VEGFR, which disrupts melanocyte function and melanin synthesis (Jin et al., 2023; Shah et al., 2021).

Despite these observations, research on sex-specific genetic mechanisms underlying ripretinib and sunitinib responses in GIST remains limited. However, genomic analyses in clear cell renal cell carcinoma (CCRCC) have revealed that female patients with elevated DKC1 expression also exhibit increased TERC levels, a pattern associated with reduced therapeutic response and shorter progression-free survival (Yuan et al., 2023). These findings suggest sex-dependent interactions between telomerase-related genes and tyrosine kinase inhibitor efficacy. Given the documented gender disparities in clinical ADRs among GIST patients—particularly cutaneous and ocular toxicities—comprehensive investigations into sex-specific molecular mechanisms are warranted. Key areas include kinase resistance pathways, telomerase regulatory networks, and polymorphisms affecting drug metabolism. Such research is essential to elucidate the biological underpinnings of therapeutic heterogeneity and to inform precision oncology through gender-stratified treatment strategies. A comparative mechanistic analysis further distinguishes ripretinib and sunitinib. Ripretinib is a novel type II switch control tyrosine kinase inhibitor designed to broadly inhibit both primary and secondary KIT and PDGFRA mutations associated with the progression of GIST (28, 29). Ripretinib exhibits high potency against the inactive form of receptor tyrosine kinases (RTKs) by binding to the switch pocket and activation loop, stabilizing the protein in an inactive conformation and thereby inhibiting its active state. This mechanism makes ripretinib a “switch control inhibitor,” with broad inhibitory activity against various secondary mutations, including KIT exons 13 (V654A), 14 (T670I), 17 (D816), and 18 (A829P) (Yu et al., 2021; Janku et al., 2020). The drug was approved by the U.S. FDA in May 2020 for patients with advanced GIST who have failed at least three prior kinase inhibitors. Relevant studies support that ripretinib provides clinical benefits at a daily dose of 150mg, with better safety and patient tolerability compared to other treatments (Zalcberg).

Through a comprehensive analysis of ripretinib-related AEs in the FAERS database, we observed that the adverse signals associated with ripretinib primarily involved categories such as gastrointestinal disorders, general disorders and administration site reactions, and skin and subcutaneous tissue diseases. These results are consistent with the common adverse events reported in ripretinib’s drug labeling and clinical trials, such as nausea, vomiting, and fatigue (Dhillon, 2020). Ripretinib predominantly induces ADRs reflecting significant tumor volume changes and vascular alterations, such as hepatic embolization, which might be attributed to the profound vascular disruption caused by VEGFR pathway inhibition (Roskoski, 2021). Tumor compression, excision, and associated pain likely reflect the drug’s effective antitumor activity leading to rapid tumor cell necrosis, edema formation, and structural deformation, necessitating medical interventions (Schwartz, 2022; Lostes-Bardaji et al., 2021). Furthermore, this study identified some high-signal adverse events not mentioned in the drug’s prescribing information, such as skin papillomas, melanocytic nevi, and blood iron reduction. These new findings indicate that ripretinib may have potential effects on the skin, blood, and metabolism, which clinicians should monitor closely (Mühlenberg et al., 2024). In addition, the study also identified signals for adverse events associated with severe outcomes. Among the reported serious adverse outcomes of ripretinib, 18.2% required hospitalization, and 10.6% involved deaths, some of which may be related to disease progression caused by the tumor. However, the possibility of severe reactions triggered by the drug itself cannot be ruled out. Notably, rare but high-signal adverse events, such as hepatic neoplasm, were reported, which are not explicitly described in the prescribing information. Their biological mechanisms and clinical significance require further research and validation.

Conversely, AEs of sunitinib prominently features distinct pathologic conditions such as diffuse uveal melanocytic proliferation, connective tissue neoplasm, and salivary gland fistula. Ocular adverse events associated with sunitinib therapy necessitate increased clinical vigilance. A case report described a male patient with renal cell carcinoma who developed bilateral diffuse uveal melanocytic proliferation (BDUMP) during sunitinib treatment, resulting in a fatal outcome (Parakh et al., 2022). BDUMP is a rare paraneoplastic ocular syndrome, typically occurring in patients with advanced, often occult, systemic malignancies (Tong et al., 2021). It serves as both a poor prognostic indicator and a marker of disease progression. The pathogenesis is hypothesized to involve ectopic production of growth factors or hormones that exert paracrine effects on distant ocular tissues. Experimental studies have demonstrated that IgG-enriched plasma fractions contain cultured melanocyte elongation and proliferation-stimulating factors (CMEP), which induce abnormal proliferation of choroidal melanocytes and retinal pigment epithelial cells (Hu et al., 2023). These pathological changes disrupt retinal pigment epithelium function, ultimately compromising the outer blood-retinal barrier (Przeździecka-Dołyk et al., 2020; Sarkar et al., 2022). Additionally, unique ocular AEs like and eyelash discoloration could result from disrupted melanocyte function and pigmentary alterations secondary to VEGFR inhibition.

Clinically, these differential AEs underscore the necessity of tailored patient risk assessment and individualized monitoring strategies. For ripretinib, clinicians should closely monitor hepatic function, tumor-related symptoms, and dermatologic health. For sunitinib, rigorous ophthalmologic evaluations, renal function monitoring, and attention to pigmentation changes are recommended, particularly in long-term therapy.

This study has several limitations. First, the FAERS database is a spontaneous reporting system that relies on voluntarily submitted adverse event reports. This reliance on spontaneous reporting may lead to underreporting of mild or common events or unusual events may be overreported. Second, the underlying conditions treated with certain drugs may predispose patients in GIST, acting as a confounding factor. Furthermore, establishing causality in pharmacovigilance and observational cohort studies is inherently challenging due to the lack of complete information in FAERS cases, such as dosage, frequency, duration of exposure, patient comorbidities, onset times, and other critical clinical details. This missing information limits the ability to fully analyze potential associations. Consequently, although we achieved a comprehensive analysis of ripretinib’s adverse reactions by removing indication restrictions, it should be noted that this generalized analytical approach may introduce data from non-target indications, potentially creating biases in identifying adverse reaction characteristics specific to GIST patients.

## 5 Conclusion

This study provides a comprehensive evaluation between ripretinib and sunitinib based on FAERS data. Known AEs were validated, and new potential safety signals were identified. The common AEs and differences between ripretinib and sunitinib should be taken into account to adjust clinical medication strategies. These findings contribute to the optimization of ripretinib and sunitinib’s clinical use and emphasize the importance of continued pharmacovigilance.
