# Correction: Gastrointestinal stromal tumors with the use of ripretinib and sunitinib: real-world adverse event analysis based on the FDA adverse event reporting system (FAERS)

**DOI:** 10.3389/fphar.2025.1689499

**Published:** 2025-09-09

**Authors:** Xinzhen Che, Yong Zhu

**Affiliations:** ^1^ First Clinical Medical College, Shandong University of Traditional Chinese Medicine, Jinan, Shandong, China; ^2^ Shandong University of Traditional Chinese Medicine Affiliated Hospital, Jinan, Shandong, China

**Keywords:** ripretinib, sunitinib, adverse events, FAERS, GIST treatment

The published article was incorrectly categorized as a Review article. The correct article type is “**Original Research**”. The original article has been updated.

